# Optimization and prediction of antioxidant properties of a tea-ginger extract

**DOI:** 10.1002/fsn3.237

**Published:** 2015-04-27

**Authors:** Solomon Akinremi Makanjuola, Victor Ndigwe Enujiugha, Olufunmilayo Sade Omoba, David Morakinyo Sanni

**Affiliations:** 1 Department of Food Science and Technology, Federal University of TechnologyAkure, Nigeria; 2 Department of Biochemistry, Federal University of TechnologyAkure, Nigeria

**Keywords:** Antioxidant, color, hue index, multivariate regression, response surface methodology, tea-ginger extract

## Abstract

A response surface approach was used to investigate the effects of temperature, concentration, and time on the antioxidant properties (total flavonoid (TF), total phenol (TP), peroxide scavenging activity (PS), iron chelating activity (IC), DPPH radical-scavenging ability (DPPH), ABTS assay (ABTS)) of aqueous extract of tea-ginger (2:1) powder. Color indices, pH, and redox potential of the tea-ginger powder were also measured and used as independent variables for the prediction of antioxidant properties of the extract using ordinary least square (OLSR), principal component (PCR), and partial least square (PLSR) regression. The *R*^2^ values for TP, TF, ABTS, and PS response surface models were 0.8873, 0.9639, 0.6485, and 0.5721, respectively. The OLSR, PCR, and PLSR were able to provide predictive models for DPPH, TP, and TF of the tea-ginger extract (*P* < 0.05). The PLSR gave the most parsimonious model with an *R*^2^ of 0.851, 0.736, and 0.905 for DPPH, TP, and TF, respectively.

## Introduction

Tea drink is widely accepted and second most consumed drink worldwide after water (Venditti et al. 2010; Carloni et al. 2013). Tea has been shown to possess several health benefits which could be attributed to its content of polyphenols. Tea polyphenols include catechins, myricetin, and quercetin. Tea catechins are efficient free radical scavengers due to their one-electron reduction potential (Ananingsih et al. 2013). Catechins have antioxidative, anticarcinogenic, antimicrobial, antiviral, anti-inflammatory, and antidiabetic properties (Ananingsih et al. 2013).

Ginger is also a very popular plant with worldwide acceptance. It has found various uses as spices, nutraceutical and pleasure drink. The health benefits of ginger are attributed to their polyphenols. The ginger polyphenols include gingerols, shogaols, and catechins. Ginger has been identified as an herbal medicinal product with pharmacological effect (Shirin and Jamuna 2010). Ginger possesses anti-atherogenic and antihypertensive property (Bhandari et al. 1998; Ghayur et al. 2008).

Since tea and ginger are widely accepted there is possibility of producing a functional drink by combining tea and ginger extracts. A combination of different redox-active compounds (antioxidants) may be needed for proper protection against oxidative stresses (Halvorsen et al. 2006) due to the synergistic benefits that could be obtained (Karna et al. 2011). A cardioprotective functional drink containing apple, blueberry and cranberry juice, and ginger extract has been formulated by Gunathilake et al. (2013). Adesokan et al. (2013) reported that the sensory attributes of *Hibiscus sabdariffa* drink were enhanced by the use of ginger and garlic. This suggests that combination of plant extracts from different sources not only produces synergistic effect in terms antioxidant activity but also in terms of sensory attributes of the resulting product.

Quality control is an essential part of any drink production factory to prevent product deviation from the specified standard. Rapid quality control techniques are essential today to keep pace with the large volume of products being churned out of the factory. The quality control techniques must not only be rapid but also affordable and clean. Color, pH, and redox potential are properties of food that can be rapidly estimated with little or no need for extensive sample preparation. These properties of food are also expected to change with process conditions, thus they could be exploited for rapid estimation of the antioxidant content of tea-ginger drinks. Makanjuola et al.(2010) reported that canned whole tomatoes packed in CaCl_2_ juice were lighter than tomatoes packed in ordinary juice. Color is an important attribute in shelf life and product quality determinations; because color analysis is nondestructive, and is easy and quick to perform, color parameters are often monitored alone or in tandem with chemical concentrations of a target compound (Li et al. 2013). Li et al. (2013) developed a mathematical model for the color degradation kinetics of epigallocatechin gallate (EGCG). EGCG is a major catechin in tea.

The popular methods for assay of antioxidant capacity are ferric reducing/antioxidant power (FRAP), 2,2-azino-bis (3-ethylbenz-thiazoline-6-sulfonic acid) (ABTS) or Trolox equivalent antioxidant capacity (TEAC), 2,2-diphenyl-1- picrylhydrazyl (DPPH), and oxygen radical absorbance capacity (ORAC). It is recommended that at least two, and preferably all of these assays be combined if possible, so as to provide comprehensive information on the total antioxidant capacity of a foodstuff, taking into account the pros and cons of each assay as well as their applicability (Pérez-Jiménez et al. 2008). As an example in the analysis of antioxidant capacity of walnut, red grape pomace and fucoidan, red grape pomace had the greatest antioxidant capacity in FRAP, ORAC, and DPPH assays, while walnut had the greatest antioxidant capacity in the ABTS assay (Pérez-Jiménez et al. 2008). The antioxidant capacities of herbal extracts have also been shown to rank differently in TEAC and DPPH assays (Tsai et al. 2008).

In this study, we seek to investigate the influence of process parameters (temperature, concentration, and time) on the antioxidant content of tea-ginger extract and also present rapid quality control techniques for estimation of the antioxidant content of the tea-ginger extract.

## Materials and Methods

### Plant material and processing

Tea (*Camellia sinensis*) leaves and ginger (*Zingiber officinale*) rhizomes were processed as described by Makanjuola et al. (2015). The tea leaves were processed into black tea. Tea leaves obtained from Obudu Mountain in Cross River state in Nigeria were sun-dried, ground, and passed through a 1.4 mm sieve. Ginger rhizomes were obtained from Kaduna state in Nigeria. The ginger rhizomes were peeled, sundried, and ground. The tea and ginger samples were passed through a 1.4 mm sieve. The obtained powders were wrapped in aluminum foil and stored under refrigerated condition (4°C) for further analysis.

### Extraction

A classical extraction was employed. The extraction was done in a conical flask placed on temperature-controlled magnetic stirrer (UC 152; Bibby Scientific, Staffordshire, UK). The stirrer speed was set at scale 3. Water was then introduced into the conical flask. The flask was covered with aluminum foil to minimize light penetration. To ensure the accuracy of the extraction temperature, a temperature controller (SCT 1, Bibby Scientific, Staffordshire, UK.) was placed inside the conical flask and connected to the temperature-controlled magnetic stirrer. Once the required extraction temperature was reached, the required weight of blended powder sample of tea-ginger (2:1) was introduced into the conical flask. Tea-ginger (2:1) powder was selected after some preliminary investigation which revealed that the tea-ginger (2:1) extract had a higher total flavonoid content compared to the tea-ginger (1:1) and tea-ginger (1:2) extracts. The extraction was continued until the required extraction time was achieved. The extract was then filtered to remove the residues.

### Response surface methodology

A face-centered central composite design with three independent variables was used. The design consisted of 20 experimental runs: eight factorial points, six axial points, and six central points. The range of the independent variables investigated were: extraction temperature (TEM: 30–96°C), powder to solvent ratio (CON: 0.12–2.10 g per 100 mL), extraction time (TIM: 5–90 min). The response variables were antioxidant properties of the extracts. The antioxidant properties were: total flavonoid content (TFC), total phenol content (TPC), ABTS radical activity, DPPH radical activity, peroxide scavenging activity (PSA), and iron chelating activity (ICA). Data were fitted to different models. RSM models considered were linear, two-factor interaction and quadratic. Analysis of variance (ANOVA) was carried out to choose the best model. The best model that was chosen was further subjected to backward regression to remove redundant variables. Both single response and multiresponse optimization were done using the desirability concept. The optimization was set to maximize all the antioxidant properties and the process conditions were set to be within the experimental range. The antioxidant properties were all given an equal weighting of one for the optimization. The quality of the model was evaluated using the lack-of-fit, the coefficient of determination (*R*^2^), adjusted *R*^2^, predicted *R*^2^, and adequate precision.

### Prediction of antioxidant properties from color and absorbance property of the extract

Color (CIE L*, a*, b*) and sample absorbance at 510 nm (A510) and 610 nm (A610) of the extracts were determined. From a* and b* values, the hue and chroma of the extracts were calculated. The hue index value was also estimated from A510 and A610. Hue index has been used in the caramel industry as an indicator of its color (Kamuf et al. 2003). The suitability of hue index in evaluating color of tea has also been reported (Goodner and Wampler 2008). A multivariate regression analysis was carried out on the obtained data. The dependent variables were the antioxidant properties. The independent variables were: L*, a*, b*, hue, chroma, A510, A610, A510/A610, and hue index. The multivariate statistics used were: ordinary least square regression (OLSR), principal component regression (PCR), and partial least square regression (PLSR). The data were scaled and centered before running the regression analysis. In the PCR analysis, the regression was run for components that explained between 90% to 99% of the variation in the independent variables. The dependent variables were also subjected to some transformation (log_10_, square root, and inverse square root) to check for improvement in the quality of the model.

### Antioxidant analysis

ABTS was determined using the method of Miliauskas et al. (2004), as described by Spradling (2008). Phosphate buffer solution (PBS) was prepared by mixing 95 mL of sodium phosphate monobasic (2.98 g per 100 mL) and 405 mL of sodium phosphate dibasic (15.6 g per 500 mL), followed by 8.04 g of sodium chloride and filled to volume (1 l). The pH was adjusted to 7.4 with 2 mol/L NaOH. ABTS stock solution was prepared by mixing 44.8 mg of ABTS, 8.12 mg potassium persulfate, and 20 mL of distilled water. The solution was allowed to react in the dark for 12 h. An ABTS working solution was prepared by mixing 5 mL of the ABTS mother solution with 145 mL of PBS. Trolox was used as standard. To 2900 *μ*L of the ABTS working solution, 100 *μ*L of each extract or standard was added and allowed to react for 15 min before reading spectrophotometrically at 734 nm against a blank solution.

DPPH was evaluated using the method of Sompong et al. (2011). The reaction mixture consisted of 1.5 mL DPPH working solution (4.73 mg of DPPH in 100 mL ethanol analytical-grade) and 300 *μ*L extract. The mixture was shaken and left to stand for 40 min in the dark at room temperature. The absorbance was read at 515 nm relative to a control (as 100%) using a spectrophotometer. The percentage of radical-scavenging ability was calculated by using the formula:

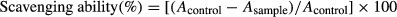
1where *A*_control_ = Absorbance at 515 nm of control, *A*_sample_ = Absorbance at 515 nm of sample.

Iron chelating activity was determined by the method of Dinis et al. (1994) as described by Ozena et al. (2011). The samples were added to a solution of 2 mmol/L FeCl_2_ (0.05 mL). The reaction was initiated by the addition of 5 mmol/L ferrozine (0.2 mL) and the mixture was incubated at room temperature for 10 min. The absorbance of the solution was measured at 562 nm. The iron chelating activity was calculated by the given formula:

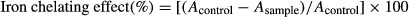
2where *A*_control_ = Absorbance at 562 nm of control, *A*_sample_ = Absorbance at 562 nm of sample.

Peroxide scavenging activity was evaluated using the method of Smirnoff and Cumbes (1989) as described by Ozena et al. (2011). Peroxide radicals were produced by mixing of FeSO_4_ and H_2_O_2_. The reaction mixture contained 1 mL FeSO_4_ (1.5 mmol/L), 0.7 mL H_2_O_2_ (6 mmol/L), 0.3 mL sodium salicylate (20 mmol/L), and appropriate volume of extracts. This mixture was incubated for 1 h at room temperature. The absorbance of the hydroxylated salicylate complex was measured at 562 nm. The percentage peroxide scavenging effect was calculated as:

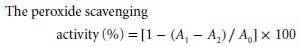
3where *A*_0_ is the absorbance of the control (without extract or standards), *A*_1_ is the absorbance including the extract or standard, and *A*_2_ is the absorbance without sodium salicylate.

Total flavonoid content was determined using the method of by Prommuaka et al. (2008). A 0.5 mL of the extracted samples or catechin solutions was mixed with 1.5 mL of 95% ethanol (v/v), 0.1 mL of 10% aluminum chloride – AlCl_3_.6H_2_O (m/v), 0.1 mL of 1 mol/l of potassium acetate, and 2.8 mL of distilled water, and the mixture was incubated at room temperature for 30 min. Absorbance of the mixture was then measured against a blank using a spectrophotometer at 415 nm. Catechin was used as standard. The blank contained all the reagents except the extract.

Total phenol content was evaluated as described by Waterhouse (2003), using the method of Slinkard and Singleton (1977). A 50 *μ*L sample of the calibration solution, extract, or blank, was taken and added to 1.58 mL water, and 100 *μ*L of Folin–Ciocalteu reagent. After 8 min, 300 *μ*L of sodium carbonate solution was added. The solutions were left at room temperature for 1 h and absorbance of each solution was determined at 765 nm against a blank. The sodium carbonate solution was prepared by dissolving 200 g of anhydrous sodium carbonate in 800 mL of water and brought to boil. After cooling, a few crystals of sodium carbonate powder were added. The solution was filtered after 24 h and made up to 1 l. Gallic acid was used as standard.

### Color and hue index analysis

Color was evaluated with a spectrophotometer CM-700d (Konica Minolta Sensing). The spectrophotometer was calibrated against a white plate. The extract was placed in a cuvette and the reading was taken. The CIE L*, a*, and b* values were read from the spectrophotometer. Readings were taken in triplicate. Hue was calculated as *θ* using eq. 4.

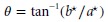
4

The following transformations were applied to the calculated *θ* McGuire (1992);

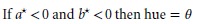
5

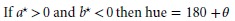
6

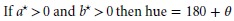
7

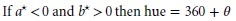
8

Chroma was calculated with eq. 9.

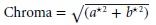
9

The hue index was calculated from eq. 10.

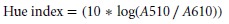
10

The A610 and A510 values were determined by measuring the absorbance of the extract against a distilled water blank in a spectrophotometer.

### Software

The response surface analysis was carried out using Design Expert v 7.0.0 (Stat-Ease, Minneapolis, USA). The multivariate statistics were done with XLSTAT Pro, 2013 (Addinsoft, Paris, France).

## Results and Discussion

### Single response optimization

Response surface methodology (RSM) is used to study how variation in independent variables affects the dependent variables with the aim of optimizing the process conditions. In this study, we investigated the effect of temperature, concentration, and time on the antioxidant properties of tea-ginger 2:1 extract. Temperature, concentration, and quadratic effect of concentration, significantly affected (*P* < 0.05) polyphenol content of the extract (Table1). Temperature, concentration, time, temperature-concentration interaction, and quadratic effects of temperature and time significantly affected flavonoid content of the aqueous extracts of the tea-ginger blend. Concentration, time, concentration–time interaction, and quadratic effect of time had significant effects on ABTS radical-scavenging activity. Concentration, time, and quadratic effects of concentration and time had significant effects on peroxide scavenging activity of the tea-ginger extract. The *R*^2^ values for total phenol content, total flavonoid content, ABTS radical-scavenging activity, and peroxide scavenging activity were 0.8873, 0.9639, 0.6485, and 0.5721, respectively. These models also have insignificant lack of fit (*P* > 0.05). Very low *R*^2^ values were obtained for DPPH radical-scavenging activity and iron chelating activity. Concentration had the highest regression coefficient in all the significant models obtained in this investigation. This points to the fact that concentration had more impact on the antioxidant properties of tea-ginger extract compared to temperature and time. A similar pattern was observed in the work of Xu et al. (2013), where concentration had the highest significant influence compared to extraction temperature and time; on the polyphenol content and FRAP values of extracts obtained during the optimization of extraction conditions for tea (*Camellia sinensis* L.) fruit peel.

**Table 1:** Response surface model for aqueous extraction of tea-ginger 2:1 powder

Source	Total flavonoid content (mg CE/L)	Total phenol content (mg GAE/L)	ABTS (mg TE/L)	Peroxide scavenging activity (%)	Iron chelating activity (%)	DPPH (%)
Transformation	Sqrt(TF)	Log_10_(TP)				
INTERCEPT	−3.3900	2.5773	0.7858	36.9479	89.1034	11.1457
TEM	1.1710	1.7622E-3				
CON	11.0702	0.6350	0.05296	31.1367		
TIM	0.4244		4.2216E-3	−0.75464		
TEM^*^CON	0.2970					
TEM^*^TIM						
CON^*^TIM			−1.063E-3			
TEM^2^	−9.4820E-3					
CON^2^		−0.1726		−12.1169		
TIM^2^	−4.2766E-3		−2.534E-5	8.5620E-3		
Model (p-value)	<0.0001	<0.0001	0.0023	0.0091		
Lack of Fit	0.4347	0.6804	0.2478	0.9832	0.5713	0.8670
R^2^	0.9639	0.8873	0.6485	0.5721	0	0
Adj R^2^	0.9472	0.8662	0.5547	0.4580	0	0
Pred R^2^	0.9018	0.8106	0.1755	0.3273		
Adeq Precision	23.406	17.274	8.203	9.464		

TEM, temperature; CON, concentration; TIM, time; Adj *R*^2^, adjusted *R*^2^; Pred *R*^2^, predicted *R*^2^; Adeq Precision, adequate precision; Sqrt(TF), square root of total flavonoid; Log_10_(TP), Log of total phenol.

**Figure 1:**
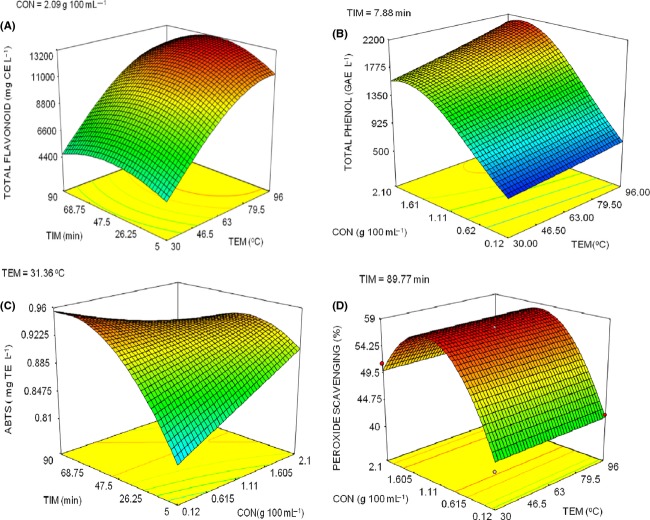
Response surface graphs showing effect of extraction variables on antioxidant properties (A), total flavonoid content (B), total phenol content (C), ABTS (D), and peroxide scavenging activity; during aqueous extraction of tea-ginger powder.

The total flavonoid content of the tea-ginger ginger extract increased rapidly as the extraction temperature increased from 30°C to 80°C and moderately increased as the extraction temperature increased from 80°C to about 92°C before a decline in the flavonoid concentration was observed (Fig.1A). The effect of extraction time on the flavonoid extraction was lower compared to that of the extraction temperature. The change in extraction time from 5 to 54 min at concentration of 2.10 g per 100 mL and temperature of 96°C led to a about 20% increase in the total flavonoid content, whereas a change in extraction temperature from 30°C to 96°C at same concentration of 2.10 g per 100 mL at an extraction time of 54 min brought about a 209% increase in the total flavonoid content of the extract (Fig.1A). There was a rapid increase in the total phenol content of the extract as the powder concentration moved from 0.12 to 1.61 g per 100 mL and then followed by a slight increase in the total phenol content to a powder concentration of about 1.9 g per 100 mL before a decline in concentration was noticed (Fig.1B). A probable reason for the reduction in the extraction yield at very low solvent to powder ratio (high powder concentration) could be that increasing the solid mass leads to a decrease in the surface area available for the solvent to penetrate the substrate and solubilize the target molecules (Destandau et al. 2013). An increase in the ABTS radical-scavenging activity of the extract was observed as the concentration of the powder increased from 0.12 to 2.10 g per 100 mL at extraction time of 5 min. The ABTS radical-scavenging activity of the extract also experienced a sharp increase as extraction time increased from 5 to about 70°C at a powder concentration of 0.12 g per 100 mL (Fig.1C). At powder concentration above 0.615 g per 100 mL, a reduction in ABTS radical scavenging activity of the extract was observed at a longer extraction time (Fig.1C). The peroxides scavenging activity of the extract increased as the powder concentration moved from 0.12 to 1.4 g per 100 mL (Fig.1D). At concentration above 1.4 g per 100 mL, a decline in the peroxide scavenging activity was observed.

Tea leaves (*Camellia sinensis*) and ginger rhizomes (*Zingiber officnale*) have high antioxidant potential. Studies in our laboratory showed that tea leaves have ABTS radical-scavenging activity, peroxide scavenging activity, iron chelating activity, and DPPH radical-scavenging activity of 0.90 mg TE/L, 82.50%, 90.62%, and 37.16%, respectively, when extracted under optimized extraction conditions. Also ginger had ABTS radical scavenging activity, peroxide scavenging activity and iron chelating activity of 0.92 mg TE/L, 73.49%, 65.30%, and 69.72%, respectively, when extracted under optimized conditions. (unpublished data). The maximum values for the antioxidant properties of tea-ginger extract obtained from the response surface plots (Fig.1) were: 13227.8 mg CE/L (total flavonoid content), 2140 mg GAE/L (total phenol content), and 0.96 mg TE/L (ABTS) and 57.56% (peroxide scavenging activity). The maximum values for the antioxidant properties of ethanolic tea-ginger 2:1 extract obtained from response surface plots (Makanjuola et al. 2015) were: 1.00 TE/L (ABTS), and 78% (peroxide scavenging activity)**.** This result indicated that ethanolic extract of tea-ginger 2:1 extract has higher ABTS radical scavenging activity and peroxide scavenging activity compared to the aqueous extract. However, the merit of the aqueous extraction of tea-ginger 2:1 powder is that it presents a lesser food safety concern compared to the ethanolic extraction. The aqueous extraction of flavonoids from the tea-ginger 2:1 powder was maximized at a temperature, concentration, and time of, 91.45°C, 2.09 g per 100 mL, 53.99 min, respectively (Figure 1A). The extraction of polyphenols was maximized at a temperature of 96°C and a concentration of 1.84 g per 100 mL (Figure 1B). The ABTS radical-scavenging activity was maximized at a concentration of 0.12 g per 100 mL and a temperature of 80.80 min (Figure 1C). A concentration of 1.27 and temperature of 89.77 min was required to maximize the peroxide scavenging activity of the aqueous extraction of tea-ginger powder (Figure 1D). A high temperature was required to maximize the extraction of flavonoid and polyphenols from the tea-ginger blend in this study. Some studies done on aqueous extraction of tea or ginger have indicated that a high temperature is required to optimize the extraction of their polyphenols. A study of the kinetics of extraction of the antioxidants from tea indicated that the best combinations of temperature and time extraction with water were 20–40 min, 80°C for epigallocatechin, epicatechin, and caffeine, and 80 min, 90°C for catechin, epicatechin gallate, and epigallocatechin gallate (Ziaedini et al. 2010). In the study of Perva-Uzunalic et al. (2006), the aqueous extraction of tea catechin, a major flavonoid in tea was favoured by high temperature extraction temperature of 80°C for 20 min or 95°C for 10 min. Gunathilake and Rupasinghe (2014), extracted fresh ginger rhizomes using hot water extraction. They reported optimum extraction condition for the ginger polyphenols will be at a temperature above 60°C and a time greater than 60 min.

**Table 2:** Confirmation runs under multiresponse optimization conditions

Response	Prediction	95% CI low	95% CI high	Validation
DPPH (%)	11.15^1^	5.06	17.23	26.73 ± 1.75
Total Phenol (mg GE/L)	2118.74	1789.90	2508.74	1783.57 ± 128.79
Total Flavonoid (mg CE/L)	8400.71	7037.06	9910.72	9320.83 ± 956.74
ABTS (mg TE/L)	0.89	0.85	0.93	0.89 ± 0.012
Peroxide Scavenging (%)	56.50	48.07	64.91	72.96 ± 1.15
Iron Chelating (%)	89.10^1^	87.61	90.60	83.84 ± 2.84

CI, Confidence interval. *n* = 3.

1 Mean values were used as the prediction for DPPH radical scavenging and iron chelating activity, since no appropriate response surface model was found for them.

### Multiresponse optimization

**Figure 2:**
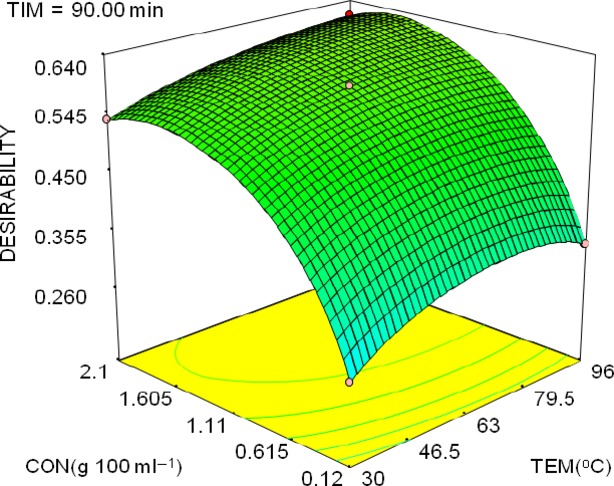
Response surface graph showing multi-response optimization condition for antioxidant extraction from tea-ginger (2:1) powder using water.

The different antioxidant properties require different optimum conditions to maximize them (Fig.1). This could lead to a situation such that as one of the antioxidant properties is being maximized another antioxidant property is being minimized. Hence, the need to employ a multiresponse optimization approach to maximize all the antioxidant properties of the tea-ginger extract. A temperature of 95.99°C, powder concentration of 1.68 g per 100 mL and extraction time of 90 min was required for the multiresponse optimization of aqueous extraction of antioxidant from tea-ginger powder (Fig.2). A decline in multiresponse desirability was observed at concentration above 1.68 g per 100 mL. The temperature used for the confirmation run was approximated to the nearest whole number by taking into account the operating convenience of the temperature controller. The values obtained from the confirmation runs (except for DPPH radical scavenging activity and peroxide scavenging activity) fell within the confidence interval values (Table2), which give an indication of the expected process average. The values of DPPH and peroxide scavenging activity were higher than the expected 95% CI high. However, since the goal of the multiresponse optimization is to maximize these antioxidant activities, these values were acceptable. The approximation of the extraction temperature to the nearest whole number may be responsible for the higher values of the DPPH radical and peroxide scavenging activities in the experimental run when compared to the expected prediction values. The higher value of the experimental DPPH radical scavenging activity when compared to the prediction value could also be due to the inability to obtain an appropriate response surface model, thus the mean value was used as the prediction target.

### Prediction models for estimation of antioxidant of aqueous tea-ginger (2:1) extract

This study employed OLSR, PCR, and PLSR to develop models that could explain the relationship between the antioxidant properties of tea-ginger extract and rapid instrumental analysis (color, hue index, pH, redox potential) – with the aim of using them to establish rapid quality control protocol for the tea-ginger extract. The OLSR and PLSR resulted in models that could predict the total flavonoid content of tea-ginger extract. The PCR resulted in a model with poor predictive quality (*Q*^2^ = −0.069, Table3). The PLSR model was able to use either the A510 or A610 of the extract to explain the total flavonoid content of the tea-ginger drink (Table3). Using the A610 of the extract, we had a PLSR model with an *R*^2^, *Q*^2^, and RMSE of 0.905, 0.893, and 7.628. When the PLSR model made use of the A510 of the extract, we had a model having an *R*^2^ of 0.885, *Q*^2^ of 0.871, and RMSE of 8.376. The OLSR produced a model with a *Q*^2^ of 0.724 and RMSE of 8.767. Also the PCR could not give a good model for the prediction of total phenol content of the tea-ginger extract. However, the PLSR and OLSR were able to produce models for the prediction of total phenolic content of the tea-ginger extract.

**Table 3:** Regression parameters for antioxidant prediction in aqueous tea-ginger extract

	Components	*R* ^2^	*Q* ^2^	RMSE
Sqrt (Total flavonoid content)				
OLSR	L^*^, a^*^, b^*^, hue, chroma, pH, redox potential, A510, A610, A510/610, hue index	0.950	0.724	8.767
PCR	L^*^, A510/A610, pH, hue	0.522	−0.069	19.725
PLSR	A610 (A510)	0.905 (0.885)	0.893 (0.871)	7.628 (8.376)
Log (Total phenol content)				
OLSR	L^*^, a^*^, b^*^, hue, chroma, pH, redox potential, A510, A610, A510/610, hue index	0.864	0.529	0.124
PCR	L^*^, A510/A610, pH, hue, chroma, a^*^,	0.692	−0.055	0.146
PLSR	A510 (A610)	0.736 (0.704)	0.712 (0.672)	0.109 (0.115)
Sqrt (DPPH)				
OLSR	L^*^, a^*^, b^*^, hue, chroma, pH, redox potential, A510, A610, A510/610, hue index	0.944	0.762	0.840
PCR	L^*^, A510/A610, pH, hue	0.859	0.578	0.978
PLSR	pH, L^*^, a^*^	0.851	0.719	0.869
ABTS				
OLSR	L^*^, a^*^, b^*^, hue, chroma, pH, redox potential, A510, A610, A510/610, hue index	0.676	−0.481	0.0459
PCR	L^*^, A510/A610, pH, hue, chroma, a^*^	0.473	−0.367	0.0460
PLSR	–	–	–	–
Peroxide scavenging activity				
OLSR	L^*^, a^*^, b^*^, hue, chroma, pH, redox potential, A510, A610, A510/610, hue index	0.697	−0.462	8.290
PCR	L^*^, A510/A610, pH, hue	0.486	−0.113	7.883
PLSR	–	–	–	–
Iron chelating activity				
OLSR	L^*^, a^*^, b^*^, hue, chroma, pH, redox potential, A510, A610, A510/610, hue index	0.783	−0.012	2.287
PCR	L^*^, A510/A610, pH, hue	0.255	−0.205	3.097
PLSR	–	–	–	–

OLSR, ordinary least square regression; PCR, principal component regression; PLSR, partial least square regression; Sqrt, square root transformation of the dependent variable; Log, log transformation of the dependent variable. The component column shows the predictors present in the different regression equations.

The PLSR model was able to use either the A510 or A610 of the extract to predict its total phenol content (Table3). The A510 model (*R*^2^ = 0.736, *Q*^2^ = 0.712, RMSE = 0.109) had a better quality than the A610 model (*R*^2^ = 0.704, *Q*^2^ = 0.672, RMSE = 0.115). The OLSR model had an *R*^2^, *Q*^2^, and RMSE of 0.864, 0.529, and 0.124, respectively. The model from the OLSR had the highest *R*^2^ (0.944), highest *Q*^2^ (0.762), and lowest RMSE (0.840) for the prediction of DPPH radical scavenging activity (Table3). The PCR used the; L*, A510/A610, pH, and hue of the tea-ginger extract to explain the DPPH radical scavenging activity. This PCR model had a lower *Q*^2^ (0.578) and higher RMSE (0.978) when compared with the OLSR and PLSR. The PLSR model was able to predict the DPPH radical scavenging activity of the tea-ginger extract using the pH, L*, and a* properties of the extract. This PLSR model had a *Q*^2^ of 0.719 and RMSE of 0.869 (Table3).

The *R*^2^ of a model tends to increase with an increase in the number of variables in the model. With this situation a case may arise such that a model may have a very high R^2^ but a low predictive quality. This same pattern was observed in this study. All the OLSR models had the highest *R*^2^ (Table3). The models with the best predictive quality (highest *Q*^2^) were mostly the PLSR models (Table3). Although the OLSR models had the highest *R*^2^, the predictive performance of the OLSR models in two of the three cases (total flavonoid content, total phenol content, DPPH) discussed above was not as strong as those of PLSR models. The OLSR models were probably suffering from overfitting. According to Cozzolino (2014), if too many independent variables are used to model a response, the solution can become overfitted – as the model will become very dependent on the data set and will give poor prediction results. This demerit of OLSR is where the advantage of PCR and PLSR modeling lies. The OLSR and PLSR allows for variable compression, thus avoiding overfitting. The drawback of the PCR modeling is that some score vectors may have very little in common with the response vectors (Ergon 2014). The PLSR model is able to overcome this PCR weakness by taking into consideration the covariance of the score vectors with the response vectors (Ergon 2014).

## Conclusion

Extraction temperature, extraction time, and powder concentration influenced the antioxidant content of tea-ginger (2:1) aqueous extract. Of these three investigated process factors, the powder concentration had the highest influence on the antioxidant content of the extract. We have also shown that A610, A510, pH, L*, and a* of the extract could be useful for rapid estimation of some of the antioxidant properties of the tea-ginger extract. Amongst the three regression techniques employed in this study, PLSR produced models that are parsimonious – and in most cases with better predictive quality.

## Conflict of Interest

None declared.

## References

[b1] Adesokan IA, Abiola OP, Adigun MO, Anifowose OA (2013). Analysis of quality attributes of Hibiscus sabdariffa (zobo) drinks. Afr. J. Food Sci.

[b2] Ananingsih VK, Sharma A, Zhou W (2013). Green tea catechins during food processing and storage: a review on stability and detection. Food Res. Int.

[b3] Bhandari U, Sharma JN, Zafar R (1998). The protective action of ethanolic ginger (*Zingiber officinale*) extract in cholesterol-fed rabbits. J. Ethnopharmacol.

[b4] Carloni P, Tiano L, Padella L, Bacchetti T, Customu C, Kay A (2013). Antioxidant activity of white, green and black tea obtained from the same tea cultivar. Food Res. Int.

[b5] Cozzolino D, Granato D, Ares G (2014). The use of correlation, association and regression to analyse processes and products. Mathematical and statistical methods in food science and technology.

[b6] Destandau E, Michel T, Rostagno MA, Prado JM, Elfakir C (2013). Microwave assisted extraction. Natural product extraction: principles and applications.

[b7] Dinis TCP, Madeira VMC, Almeida LM (1994). Action of phenolic derivatives (acetaminophen, salicylate and 5-aminosalicylate) as inhibitors of membrane lipid peroxidation as peroxyl radical scavenging effects. Chem. Pharm. Bull.

[b8] Ergon R, Granato D, Ares G (2014). Principal component regression (PCR) and partial least squares regression (PLSR). Mathematical and statistical methods in food science and technology.

[b9] Ghayur MN, Gilani AH, Janssen JL (2008). Ginger attenuates acetylcholine induced contraction and Ca+2 signalling in murine airway smooth muscle cells. Can. J. Physiol. Pharmacol.

[b10] Goodner KL, Wampler B (2008). http://www.synergytaste.com/sites/synergytaste.com/files/SEN-TN-0002-Measuring_Tea_Color_Using_A_Simple_Spectrometric_Assay.pdf.

[b11] Gunathilake KDPP, Rupasinghe HPV (2014). Optimization of water based- extraction for the preparation of bioactive-rich ginger extract using response surface methodology. Eur. J. Med. Plants.

[b12] Gunathilake KDPP, Rupasinghe HP, Pitts NL (2013). Formulation and characterization of a bioactive-enriched fruit beverage designed for cardio-protection. Food Res. Int.

[b13] Halvorsen BL, Carlsen MH, Phillips KM, Bohn SK, Holte K, Jacobs DR (2006). Content of redox-active compounds (ie, antioxidants) in foods consumed in the United States. Am. J. Clin. Nutr.

[b14] Kamuf W, Nixon A, Parker O, Barnum GC (2003). Overview of caramel colors. Cereal Foods World.

[b15] Karna P, Chagani S, Gundala SR, Rida PC, Asif G, Sharma V (2011). Benefits of whole ginger extract in prostate cancer. Br. J. Nutr.

[b16] Li N, Taylor LS, Ferruzzi MG, Mauer LJ (2013). Color and chemical stability of tea polyphenol (−)-epigallocatechin-3-gallate in solution and solid states. Food Res. Int.

[b17] Makanjuola SA, Akanbi CT, Enujiugha VN (2010). Sensory characteristics and sterilization value of unpeeled whole tomato in juice. Agric. Eng. Int. CIGR J.

[b18] Makanjuola SA, Enujiugha VN, Omoba OS, Sanni DM (2015). Application of RSM and multivariate statistics in predicting antioxidant property of ethanolic extracts of tea-ginger blend. Eur. J. Med. Plants.

[b19] McGuire RG (1992). Reporting of objective color measurements. HortScience.

[b20] Miliauskas G, Venskutonis P, van Beek T (2004). Screening of radical scavenging activity of some medicinal and aromatic plant extracts. Food Chem.

[b21] Ozena T, Demirtas I, Aksit H (2011). Determination of antioxidant activities of various extracts and essential oil compositions of Thymus praecox subsp. skorpilii var, Skorpilii. Food Chem.

[b22] Pérez-Jiménez J, Arranz S, Tabernero M, Díaz-Rubio ME, Serrano J, Goñi I (2008). Updated methodology to determine antioxidant capacity in plant foods, oils and beverages: extraction, measurement and expression of results. Food Res. Int.

[b23] Perva-Uzunalic A, Skerget M, Knez Z, Weinreich B, Otto F, Gruner S (2006). Efficiency of active ingredients from green tea (*Camellia sinensis*): extraction efficiency of major catechins and caffeine. Food Chem.

[b24] Prommuaka C, De-Eknamkulb W, Shotipruka A (2008). Extraction of flavonoids and carotenoids from Thai silk waste and antioxidant activity of extracts. Sep. Purif. Technol.

[b25] Shirin AP, Jamuna P (2010). Chemical composition and antioxidant properties of ginger root (Zingiber officinale). J. Med. Plants Res.

[b26] Slinkard K, Singleton VL (1977). Total Phenol Analysis: automation and comparison with manual methods. Am. J. Enol. Vitic.

[b27] Smirnoff N, Cumbes QJ (1989). Hydroxyl radical scavenging activity of compatible solutes. Phytochemistry.

[b28] Sompong R, Siebenhandl-Ehn S, Linsberger-Martina G, Berghofer E (2011). Physicochemical and antioxidative properties of red and black rice varieties from Thailand, China and Sri Lanka. Food Chem.

[b29] Spradling VB 2008. Phenolics in Red Wine Pomace and their Potential Application In Animal and Human Health. M.Sc Thesis, Department of Food Science, University of Missouri.

[b30] Tsai TH, Tsai TH, Chien YC, Lee CW, Tsai PJ (2008). In vitro antimicrobial activities against cariogenic streptococci and their antioxidant capacities: a comparative study of green tea versus different herbs. Food Chem.

[b31] Venditti E, Bacchetti T, Tiano L, Carloni P, Greci L, Damiani E (2010). Hot vs. Cold water steeping of different teas: do they affect antioxidant activity?. Food Chem.

[b32] Waterhouse AL (2003). Determination of total phenolics. Curr. Protoc. Food Anal. Chem.

[b33] Xu P, Bao J, Gao J, Zhou T, Wang Y (2013). Optimisation of extraction of phenolic antioxidants from tea (Camellia Sinensis L.) fruit peel biomass using response surface methodology. BioResources.

[b34] Ziaedini A, Jafari A, Zakeri A (2010). Extraction of antioxidants and caffeine from green tea (*Camelia sinensis*) leaves: kinetics and modeling. Food Sci. Technol. Int.

